# Regulation of Antimicrobial Peptides in *Aedes aegypti* Aag2 Cells

**DOI:** 10.3389/fcimb.2017.00022

**Published:** 2017-02-03

**Authors:** Rudian Zhang, Yibin Zhu, Xiaojing Pang, Xiaoping Xiao, Renli Zhang, Gong Cheng

**Affiliations:** ^1^Tsinghua-Peking Center for Life Sciences, School of Medicine, Tsinghua UniversityBeijing, China; ^2^School of Life Science, Tsinghua UniversityBeijing, China; ^3^SZCDC-SUSTech Joint Key Laboratory for Tropical Diseases, Shenzhen Center for Disease Control and PreventionShenzhen, China

**Keywords:** insect immunity, innate immunity, antimicrobial peptides, mosquito, regulation

## Abstract

Antimicrobial peptides (AMPs) are an important group of immune effectors that play a role in combating microbial infections in invertebrates. Most of the current information on the regulation of insect AMPs in microbial infection have been gained from *Drosophila*, and their regulation in other insects are still not completely understood. Here, we generated an AMP induction profile in response to infections with some Gram-negative, -positive bacteria, and fungi in *Aedes aegypti* embryonic Aag2 cells. Most of the AMP inductions caused by the gram-negative bacteria was controlled by the Immune deficiency (Imd) pathway; nonetheless, *Gambicin*, an *AMP* gene discovered only in mosquitoes, was combinatorially regulated by the Imd, Toll and JAK-STAT pathways in the Aag2 cells. *Gambicin* promoter analyses including specific sequence motif deletions implicated these three pathways in *Gambicin* activity, as shown by a luciferase assay. Moreover, the recognition between Rel1 (refer to Dif/Dorsal in *Drosophila*) and STAT and their regulatory sites at the *Gambicin* promoter site was validated by a super-shift electrophoretic mobility shift assay (EMSA). Our study provides information that increases our understanding of the regulation of *AMP*s in response to microbial infections in mosquitoes. And it is a new finding that the *A. aegypti* AMPs are mainly regulated Imd pathway only, which is quite different from the previous understanding obtained from Drosophila.

## Introduction

Insects represent more than half of all known animals in the world and co-exist with numerous microorganisms in variable environments (Novotny et al., 2002; Engel and Grimaldi, 2004). Insects have evolved effective immune systems to defend themselves against microbe-caused deterioration. In contrast to the immune system in mammals, insects lack immunoglobulin-based adaptive humoral immune responses. Thus, the innate immune response plays a dominant role in combating microbial infections in insects, in which the induction of a spectrum of antimicrobial peptides (AMPs) is a general systemic immune response (Meister et al., 1997; Lemaitre and Hoffmann, 2007). AMPs are a group of short peptides that electrostatically or hydrophobically interact with bacterial surfaces to orchestrate their elimination via different mechanisms, including lysis, disruption of proton gradients, or membrane perturbations (Hancock and Sahl, 2006). Different insects express a variable spectrum of immune-induced AMPs. In *Drosophila*, 20 *AMPs* categorized into 7 groups, including 1 *Defensin* (*Def*), 4 *Cecropins* (*Cec*), 2 *Diptericins* (*Dpt*), 4 *Attacins* (*ATT*), 1 *Drosocin* (*Dro*), 7 *Drosomycins* (*Drs*) and 1 *Metchnikowin* (*Met*), have been identified in the genome (Lemaitre and Hoffmann, 2007). Parts of these genes clustered in a short area in *Drosophila* genome, and shared similar binding sites for regulatory factors so that they can be regulated in similar manners (Deng et al., 2009): Attacin, Diptericin, and Drosocin peptides effectively oppose Gram-negative bacterial infection (Wicker et al., 1990; Bulet et al., 1993; Asling et al., 1995). Defensin is a bactericidal agent against Gram-positive bacteria (Dimarcq et al., 1994), whereas Drosomycin and Metchnikowin peptides show strong antifungal activity (Fehlbaum et al., 1994; Levashina et al., 1995). The 17 *AMPs* discovered to date in *A. aegypti* are categorized into only 5 independent families, including 4 *Defensins*, 10 *Cecropins*, 1 *Diptericin*, 1 *Attacin*, and 1 *Gambicin* (*GAM*) (Xiao et al., 2014). However, no *Drosomycin, Drosocin*, or *Metchnikowin* was identified in *A. aegypti* genome (Christophides et al., 2002; Xiao et al., 2014). This large variation in the spectrum and constitution among insect *AMPs* indicates different regulatory patterns in response to microbial infections.

Insects are equipped with multiple immune signaling pathways responding microbial invasion with *AMP* production, including the immune deficiency (Imd), Toll, and Janus kinase (JAK)-signal transduction and activators of transcription (STAT) pathways (Lemaitre and Hoffmann, 2007; Waterhouse et al., 2007; Cheng et al., 2016). The mechanisms of these immune pathways have been largely elucidated in *Drosophila*. The orthologs of the core components of these pathways have been identified in the mosquito genomes (Nene et al., 2007; Waterhouse et al., 2007; Arensburger et al., 2010). It was reported that the Toll and Imd pathway utilizes multiple immune receptors to recognize Gram-positive, Gram-negative bacteria, yeasts, and fungi and initiates signaling cascades. However, the JAK-STAT pathway is one of predominant immune signaling to viral infections in mosquitoes (Souza-Neto et al., 2009; Cheng et al., 2016).

Mosquitoes are disease vectors for hundreds of human pathogens worldwide. The AMPs in the mosquitoes act as important immune effectors to prevent pathological damages that can be caused by the persistent propagations of the arboviruses or *Plasmodium* carried in their tissues (Kokoza et al., 2010; Xiao et al., 2014). Moreover, AMPs play an important role in mosquito gut immunity, which is essential to control unexpected microbial overgrowth or opportunistic infections in the gut lumen (Xi et al., 2008; Dong et al., 2009; Pang et al., 2016). However, the precise regulatory patterns of *AMPs* in mosquitoes still remain unclear. Considering the living habit and environment, we hypothesized that the downstream activity of the immune signaling pathways, and particularly the regulation pattern of AMPs might not be the same between *Drosophila* and *Aedes* mosquitoes. Here, we examine the induced spectrum of *AMPs* in *A. aegypti* Aag2 cells. Most *A. aegypti AMPs* are strongly induced by infection of some Gram-negative bacteria via the Imd pathway. Intriguingly, induction of *Gambicin* can be combinatorially regulated by the Imd, Toll, and JAK-STAT pathways in the Aag2 cells. In the *Gambicin* promoter region, the regulatory sites for these three pathways were identified and subsequently validated using a luciferase assay and an electrophoretic mobility shift assay (EMSA). This study provides information on the complicated mechanism of *AMP* regulation in mosquitoes.

## Materials and methods

### Animal, cells, and bacteria

The animal model, *Aedes aegypti* (the Rockefeller strain) was maintained in the laboratory in a low-temperature illuminated incubator (model 818, Thermo Electron Corporation, Waltham, MA, USA) at 26°C and 80% humidity according to standard rearing procedures (Xiao et al., 2014). *A. aegypti* Aag2 cells were cultured at 28°C in Schneider's *Drosophila* medium for maintenance and microbial challenge. The cell media were supplemented with 10% heat-inactivated fetal bovine serum, 2 mM L-glutamine, and 100 U/mL each of penicillin and streptomycin. The *E. coli* ST515 strain, which is equipped with a *GFP* reporter and spectinomycin resistance, was cultured on LB plates or LB medium with 100 μg/mL spectinomycin at 37°C. *B. subtilis* TH4545 strain was cultured on LB plates or LB medium with 5 μg/mL kanamycin at 37°C. *S. aureus* BAA-1696 strain was cultured on LB plates or LB medium with 25 U/mL polymyxin B at 37°C. *C. albicans* was cultured on YPD plates or YPD medium with 100 μg/mL ampicillin and 50 μg/mL kanamycin at 30°C. *S. marcescens, E. faecium* and *Leucobacter* spp. were isolated from adult mosquito midgut (Pang et al., 2016) and cultured on LB plates or LB medium without any antibiotics at 37°C.

### Construction of recombinant plasmids

The genes of *A. aegypti Rel1A* (*AAEL007696*), *Rel1B* (*AAEL006930*), and *STAT* (*AAEL009692*) were isolated from an *A. aegypti* cDNA library, and then cloned into pAc5.1-V5-His A vector (Invitrogen, Cat. No# V4110-20). The recombinant expression plasmids were named as pAc.5.1-Rel1A-V5, pAc5.1-Rel1B-V5, and pAc5.1-STAT-V5, respectively. For the truncation assay, the reporter plasmids were constructed by inserting the truncations of *Gambicin* promoter into a pGL3-Basic plasmid (Promega, Cat. No# E1751). The inserted promoter regions were followed down-stream by a *firefly luciferase* gene. A *renilla luciferase* gene was inserted into pAc5.1-V5-His A (pAc5.1-Renilla) was transfected as an internal control. The plasmids with mutants (M1–M4) were constructed by the pGL3 plasmids with 1000 bp promoter region (pGL3-1k) via a Fast MultiSite Mutagenesis System (Transgen, Cat. No# FM201).

### Gene silencing in the Aag2 cells

The monolayer cells without aggregation were suitable for transfection. Briefly, the Aag2 cells were seeded at 3 × 10^6^ cells/mL per well in a 6-well plate. The cells formed a monolayer after 12 h of culture. Then, 2 μg of dsRNA was premixed with Effectene® (Qiagen, Cat. No# 301425) according to the manufacturer's instructions, and consequently added to the cells. After 6–18 h of transfection, the medium was replaced with fresh medium. The cells were cultured for the following investigation.

### Microbial infection in the Aag2 cells

The monolayer Aag2 cells were seeded as described above. Microbe cells cultured to logarithmic phase were collected by centrifuge and washed with sterile PBS twice. The bacteria resuspended by PBS was then added into the cultured cell medium. The final microbial concentration in the cell medium was 0.05 OD_600_ for each microorganism. 12 h later, the stimulated Aag2 cells were collected to isolate total RNA that was synthesized into cDNA for qPCR detection.

### Microbial infection in animal models

Adult female mosquitoes were kept on ice for 15 min, and then transferred to a cold tray to receive a systemic injection of 300 nL of 5 OD_600_ microbial cells at logarithmic phase (Cheng et al., 2010; Liu et al., 2014; Xiao et al., 2014). 6 h later, the inoculated mosquitoes were sacrificed to isolate total RNA for quantitative AMP mRNA analysis.

### Detection of AMP expression by qPCR

The samples of animal models or cells were homogenized in Buffer I of an RNeasy Mini Kit (Qiagen, Cat. No# 74106) with a Pestle Grinder System (Fisher Scientific, Cat. No# 03-392-106). The detailed procedure of total RNA isolation is described in the RNeasy Kit manual. cDNA was randomly reverse-transcribed using an iScript™ cDNA Synthesis Kit (Bio-Rad, Cat. No# 1708891). The *AMP* expression was then relatively quantified with qPCR by SYBR® Green II method. The primers are shown in the Supplementary Table S2. The amount of AMP expression was normalized with *A. aegypti actin* (*AAEL011197*). Fold changes were calculated by comparison to the corresponding controls using the comparative Ct method (2^−ΔΔCt^). All the primer pairs used in this study were reported in the previous research (Xiao et al., 2014, 2015; Pang et al., 2016) and the specific amplification reactions were confirmed by melt curve analysis.

### Luciferase assay

The recombinant pGL3 plasmids with *Gambicin* promoter regions were mixed with pAc5.1-Renilla (19:1 w/w), and the plasmid mixture were subsequently transfected into the Aag2 cell with Effectene® Reagent (Qiagen, Cat. No# 301425). 36 h later, 0.05 OD_600_ *E. coli* cells at logarithmic phase were used for bacterial challenge for 12 h. And then, the treated cells in a well of 48-well plate (about 6.5 × 10^4^ cells) were harvested for lysis by 100 μL per well 1 × Passive Lysis Buffer (PLB) supplied with the Dual-luciferase Report System Kit (Promega, Cat. No# E1910). The lysates were centrifuged, and subsequently the supernatant was transferred into a 96-well white polystyrene assay plate (Corning, Cat. No# 3922). 50 μL of each LAR-II and Stop&Glo solution were subsequently added into the wells for detection of *firefly* and *renilla luciferase* signals. The fluorescence was detected by a Varioskan® Flash reader (Thermo-Fisher, Cat. No# 5250030). The value of firefly luciferase was normalized by that of renilla luciferase.

### Electrophoretic mobility shift assay

The EMSA is used to validate the binding of specific nuclear regulator proteins and the specific potential regulation sites in the promoter area. This assay includes two steps: Nuclear protein extraction and EMSA assay.

The Aag2 cells were transfected by the plasmids of pAc.5.1-Rel1A-V5 / pAc5.1-Rel1B-V5 (1:1 mix) or pAc5.1-STAT-V5 for 48 h, respectively. Then infected the cells with logarithmic-phase *E. coli* at a final concentration of 0.05 OD_600_ for 12 h as described above. Subsequently, the transfected and infected cells were washed twice and collected in cold PBS buffer. The cell nuclear was extracted by a NE-PER nuclear extraction kit (Thermo-Fisher, Cat. No# 78833). Briefly, the transfected cells in a well of 6-well plate (about 8 × 10^5^ cells) were lysed by 200 μL of CER-I buffer containing 1 × complete EDTA-Free protease inhibitor (Roche, Cat. No# 04 693 132 001). Then, 11 μL ice-cold CER-II was added to the tubes. After centrifugation (16,000 × g, 5 min at 4°C), the pellets were resuspended in 100 μL ice-cold NER reagent. The samples were placed on ice and continued vortexing for 15 s every 10 min for 4 times. And then, the tubes were centrifuged at 16,000 × g for 10 min. The supernatant fractions (nuclear protein extracts, NPE) were immediately transfer to a clean pre-chilled tube. The nuclear protein extracts were stored at −80°C freezer until using.

The EMSA assay was performed with a LightShift® EMSA Kit (Thermo-fisher, Cat No# 20148) according to the manufacturer's instruction. Briefly, the nuclear protein extracts, biotin-labeled oligonucleotide probes and 1 μL of anti-V5 antibody (MBL, Cat. No# M167-3, Lot. No. 005), were added into a 20 μL reaction system, including 1 × binding buffer (1 mM Tris, 50 mM KCl, 1 mM DTT; pH 7.5), 0.05% Non-idet P-40, 2.5% glycerol and 50 ng/μL poly (dI:dC). Unlabeled or mutant oligonucleotide served as the competitors. The unlabeled probe can eliminate the shift by compete the binding of the probe and the regulator protein; the mutant oligonucleotide cannot compete the binding, so they can not eliminate the shift band. After incubation at room temperature for 20 min, the reaction mixture was run on 5% polyacrylamide gel, and then the bounds were transferred to a Nylon membrane (Bio-Rad, Cat. No# 162-0153) via a Trans-blot® SD Simi-Dry Transfer System (Bio-Rad, Cat. No# 170-3940). Consequently, the DNA was cross-linked to the membrane for 15 min. The membrane was replaced in 20 mL blocking buffer for 15 min, and then additionally incubated in another 20 mL blocking buffer containing 66.7 μL stabilized streptavidin-HRP conjugate (1:300 dilution) for 1 h. After 4 washings, the membrane was transferred to a new container with 30 mL substrate equilibration buffer and incubated for 5 min. After treated by a substrate working solution provided by the EMSA kit, the membrane was imaged by a ChemiDoc™ Imaging System (Bio-Rad, Cat. No# 1708251).

### Statistics

Mosquitoes were randomly allocated into different groups. Mosquitoes that died before measurement were excluded from analysis. The investigators were not blinded to the allocation during the experiments or to the outcome assessment. All experiments were performed independently at least 2 times. Descriptive statistics are provided in the figure legends. A Kruskal–Wallis analysis of variance was conducted to detect any significant variation among replicates. If no significant variation was detected, the results were pooled for further comparison. Given the nature of the experiments and the type of samples, differences in continuous variables were assessed with the non-parametric Mann–Whitney test. Differences in *AMP* fold changes were analyzed by using *t*-test with Welch's correction. All results are expressed as mean ± S.E.M. from independent experiments. *P*-values < 0.05 were considered significant (^*^P < 0.05, ^**^P < 0.005, ^***^P < 0.0005 and ^****^P < 0.0001). All analyses were performed using GraphPad Prism® statistical software.

## Results

### Regulation of *AMP* genes in microbial infections of the Aag2 cells

The Aag2 cell line is an *A. aegypti* cell lineage of embryonic origin (Peleg, 1968). This cell lineage is immuno-competent and has similar immune responses to that of live *A. aegypti* mosquitoes. Therefore, it is widely used as an immune cell model for studies of mosquito immunity (Gao et al., 1999; Fallon and Sun, 2001; Sim and Dimopoulos, 2010; Barletta et al., 2012).

**Figure 1:**
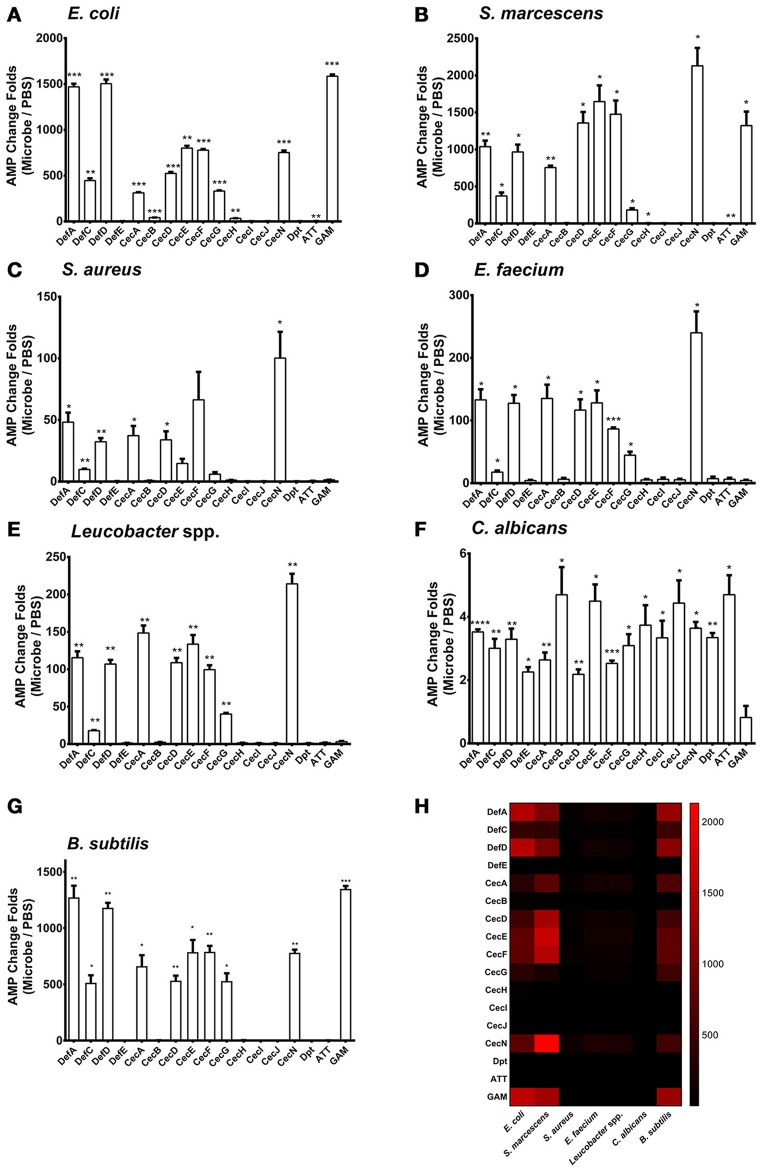
**Regulation of ***AMP*** genes in microbial infections in the Aag2 cells ***E. coli*** (A)**, *S. marcescens* **(B)**, *S. aureus* **(C)**, *E. faecium* **(D)**, *Leucobacter* spp. **(E)** *C. albicans* **(F)**, and *B. subtilis* **(G)** cells at 0.05 OD_600_ were incubated with the Aag2 cells. Uninfected cells served as controls. 12 h later, the stimulated Aag2 cells were collected to isolate total RNA that was synthesized into cDNA for *AMP* detection. The qPCR primers for each *AMP* gene are described in Supplementary Table S2. The *AMP* stimulation is presented as the fold change relative to that in the control cells without bacterial treatment. The data are presented as the mean ± S.E.M. The differences between microbes treated groups and negative control group were analyzed by using *t*-test with Welch's correction. **(H)** The changing folds were presented with a heat map to indicate the AMP induction manner between Gram negative bacteria and *B. subtilis* infection. Red means high induction as described in the scale bar. The results from 2 independent experiments were combined. ^*^p < 0.05, ^**^p < 0.005, ^***^p < 0.0005, and ^****^p < 0.0001. Def, Defensin; Cec, Cecropin; Dpt, Diptericin; ATT, Attacin; GAM, Gambicin.

We examined the patterns of *AMP* regulation in various microbe infections. We added *Escherichia coli* (Gram-negative), *Serratia marcescens* (Gram-negative), *Staphylococcus aureus* (Gram-positive), *Enterococcus faecium* (Gram-positive), *Leucobacter* spp. (Gram-positive), and *Candida albicans* (fungi) at 0.05 OD_600_ onto a confluent monolayer of the Aag2 cells, respectively. Uninfected cells served as negative controls. 12 h later, the treated cells were collected to isolate total RNA for *AMP* detection by quantitative PCR (qPCR). 3 *Defensins* (*Def A, C* and *D*), 6 *Cecropins* (*Cec A, D, E, F, G*, and *N*), and *Gambicin* were dramatically induced, while the other *AMPs* showed a modest induction or no response to the *E. coli* and *S. marcescens* infections (Figures 1A,B). A similar pattern of *AMPs* with a modest induction presents by infection with the Gram-positive bacteria *S. aureus, E. faecium*, and *Leucobacter* spp. (Figures 1C–E). However, infection with a fungi *C. albicans* rarely induced *AMP* expression in the Aag2 cells (Figure 1F). We examined the cell death after incubated with microbes. There were no significant differences between the microbes treated groups and the untreated group, suggesting that the induction of *AMP* genes is not due to a response of killed cells (Supplementary Figure S1).

We next determined the *AMP* expression pattern after infection with *Bacillus subtilis*, a Gram-positive bacterium. The regulatory spectrum of *AMPs* caused by *B. subtilis* infection was similar to that caused by Gram-negative bacterial infection (Figures 1G,H). This phenomenon may be reasoned from that *Bacillus* genus share the similar DAP-type peptidoglycans as those in Gram-negative bacteria (Nguyen-Huy et al., 1976) which enables to activate the Imd pathway in *Drosophila* (Lemaitre et al., 1997; Leulier et al., 2003). Then, we assessed the *in vivo* AMP expression pattern in live *A. aegypti* mosquitoes, 6 h following infection through intrathoracic injection of various microbes. The *in vivo* AMP expression pattern in live mosquitoes shares similarity to that of Aag2 cells (Supplementary Figure S2). This may be due to the complication of mosquito immune system in which other mechanism, such as phagocytosis, encapsulation, and complement-like factors, may be induced after infection (Hillyer and Christensen, 2002; Waterhouse et al., 2010; Barletta et al., 2012).

### Induction of AMPs is predominantly controlled by the Imd pathway in Aag2 cells

**Figure 2:**
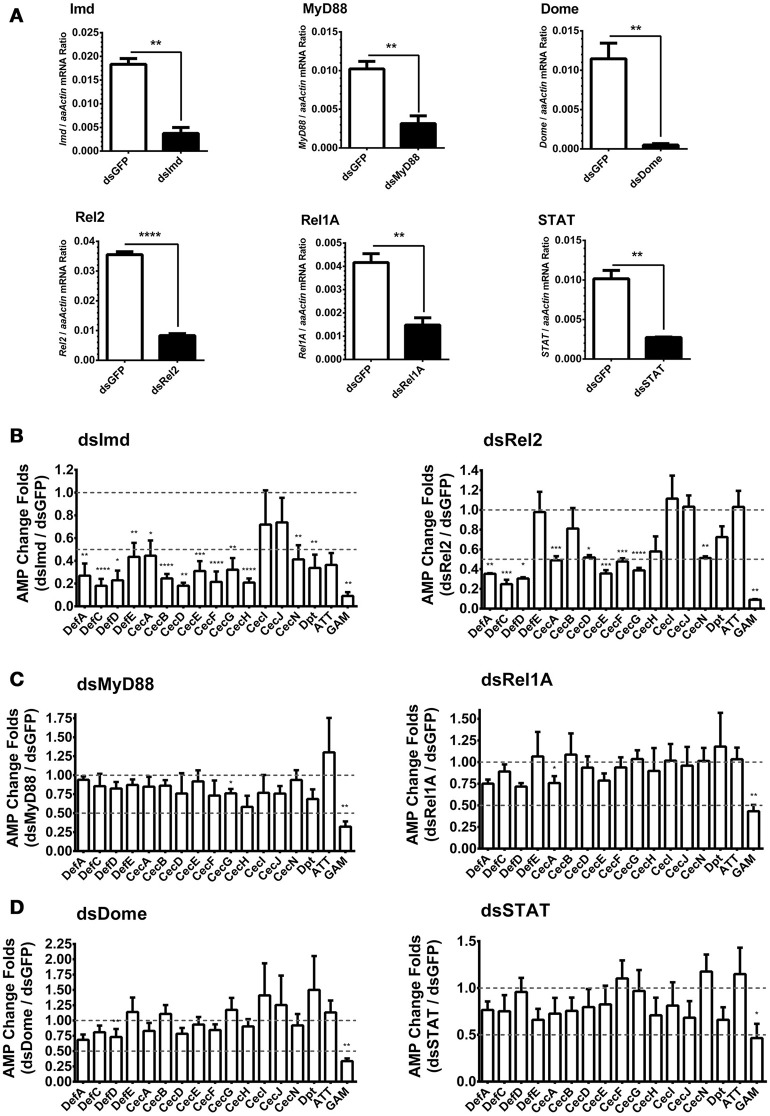
**Role of immune pathways in ***E. coli***-mediated ***AMP*** induction in Aag2 cells (A)** dsRNA-mediated silencing efficiency of key components of the immune pathways in the Aag2 cells. Expression of the key components in the Imd (*Imd* and *Rel2*), Toll (*MyD88* and *Rel1A*), and JAK-STAT (*Dome* and *STAT*) pathways were silenced by double-stranded RNA (dsRNA) transfection in the Aag2 cells. *GFP* dsRNA (dsGFP) served as control. The expression of these genes was determined by qPCR and normalized to the expression of *A. aegypti actin*. The qPCR primers are shown in Supplementary Table S2. The data were presented as the mean ± S.E.M. The data are analyzed using the non-parametric *Mann-Whitney* test. **(B–D)** Expression of the key components in the Imd (*Imd* and *Rel2*) **(B)**, Toll (*MyD88* and *Rel1A*) **(C)**, and JAK-STAT (*Dome* and *STAT*) **(D)** pathways were silenced by dsRNA transfection in the Aag2 cells. *GFP* dsRNA was used as a negative control. Subsequently, *E. coli* at 0.05 OD_600_ were incubated with the transfected cells. The *AMP* expression was then determined by qPCR 12 hrs after the bacterial challenge. The qPCR primers for each *AMP* genes are described in Supplementary Table S2. The *AMP* stimulation is presented as the fold change in inducing relative to that in the GFP dsRNA treated (mock control) cells. The data are presented as the mean ± S.E.M. The difference between the AMP induction of gene silenced groups and mock control group were analyzed by using *t*-test with Welch's correction. The results from 3 independent experiments were combined. ^*^p < 0.05, ^**^p < 0.005, ^***^p < 0.0005, and ^****^p < 0.0001. *aaActin, A. aegypti Actin*.

In *Drosophila, AMPs* are regulated by both Toll and Imd pathways. However, the regulatory mechanism of *AMPs* expression in *A. aegypti* is still unclear. We have found that infections with *E. coli, S. marcescens*, and *B. subtilis* are capable of dramatically activating *AMP* expression in Aag2 cells by a similar manner (Figure 1H). Besides, It was known that *Bacillus* bacteria activates the Imd pathway by its DAP-type peptidoglycans (Nguyen-Huy et al., 1976). We therefore determined whether the induction of *AMPs* is controlled by the Imd pathway. Expression of the key components in the Imd [*Imd* and *Rel2* (refer to *Relish* in *Drosophila*)], Toll [*Myeloid Differentiation Factor 88* (*MyD88*) and *Rel1A*], and JAK-STAT [*Domeless* (*Dome*) and *STAT*] pathways were silenced by double-stranded RNA (dsRNA) transfection in the Aag2 cells (Figure 2A). *Green fluorescent protein* (*GFP*) dsRNA was used as a negative control. Subsequently, *E. coli* at 0.05 OD_600_ was added to the transfected cells. *AMP* expression was determined by qPCR 12 h after the bacterial challenge. Knockdown of the Imd pathway components (*Imd* and *Rel2*) dramatically impaired the induction of most *AMPs* (fold change more than 2) in the Aag2 cells (Figure 2B, Supplementary Figure S3 and Supplementary Table S1); however, genetic manipulation of the Toll and JAK-STAT components showed only a modest change (fold change less than 2) for a few of the *AMP* transcripts (Figures 2C,D, Supplementary Figure S3 and Supplementary Table S1), suggesting induction of *AMPs* is predominantly controlled by the Imd pathway. In the Imd-mediated signaling cascade, the cleaved Imd activates a MAPK kinase kinase, TAK1 (transforming growth factor β-activated kinase 1), which is responsible for further activating both the JNK (c-Jun N-terminal kinase), and IKK (inhibitor of κB kinase)/Relish branches of the Imd pathway (Silverman et al., 2003; Kleino and Silverman, 2014). We therefore determined the *AMPs* regulation in the *JNK*-silencing Aag2 cells (Supplementary Figure S4A). Impairment of *JNK* expression did not show any reduction, however showed enhancement in some *AMP* transcripts (fold change more than 2), such as *Def E, Cec B, Cec I, Cec J*, and *Dpt*, in the Aag2 cells (Supplementary Figure S4B), suggesting that the JNK signaling is likely to play a negative-regulatory role in *AMP* expression in mosquitoes. Therefore, we show a different AMPs regulatory pattern between *A. aegypti* and *Drosophila*. The *Drosophila AMPs* are regulated both Toll and Imd pathway (Meister et al., 1997). However, the *AMPs* in *A. aegypti* are mostly regulated by Imd pathway (Guillen et al., 2014; Yadav et al., 2015). Nevertheless, one *AMP*–*Gambicin*–is uniquely regulated by all the three pathways in Aag2 cells, which let us to focus on this peptide.

### Induction of *Gambicin* is combinatorially regulated by the Imd, toll, and JAK-STAT pathways

**Figure 3:**
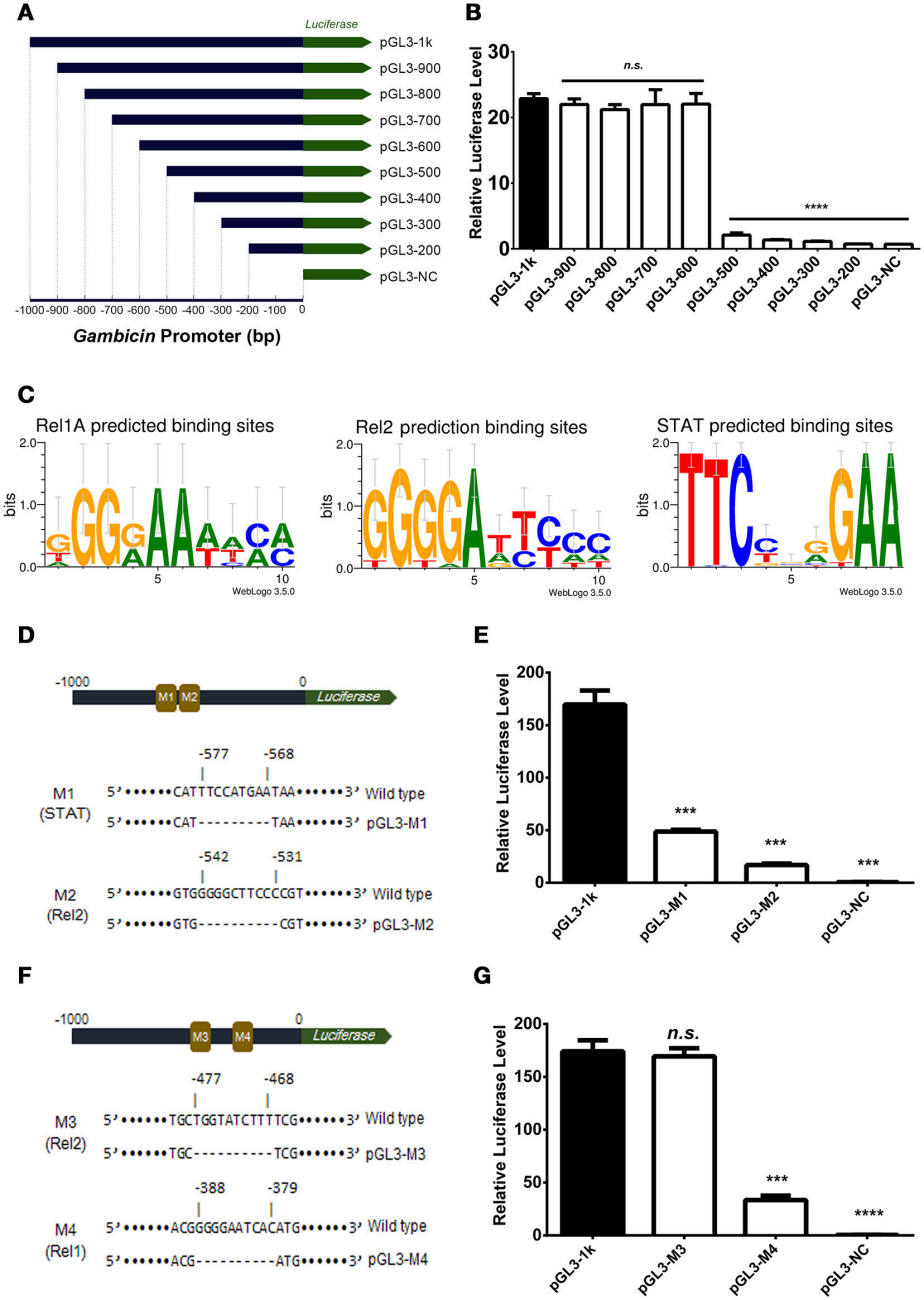
*****Gambicin*** induction is combinatorially regulated by the Imd, Toll and JAK-STAT pathways (A)** Schematic representation of the truncation design. The 1000 bp promoter region upstream of the *Gambicin* gene was cloned into a pGL3-Basic vector (pGL-1k). The truncations of the *Gambicin* promoter, which were sequentially truncated by the deletion of 100 bp segments from the 5′-end of promoter region, were inserted into the same plasmid. The inserted promoters were followed down-stream by a firefly *luciferase* gene (the green arrow), thereby enabling the determination of the regulatory activity of the inserted promoters using a luciferase assay. **(B)** Assessment of promoter activity by 100 bp sequential truncations. The recombinant plasmids with truncated *Gambicin* promoter were transfected into the Aag2 cells to determine the promoter activity via a luciferase assay. **(C)** Characterization of the regulatory sites of the Rel1, Rel2 and STAT transcription factors. The regulatory sites were predicted by WebLogo 3.5.0 (http://weblogo.threeplusone.com/create.cgi) and Vector NTI Advanced® 11.5.1 software (Invitrogen, US) with the threshold of 85%. **(D)** Schematic representation of M1 (STAT) and M2 (Rel2) mutants in the *Gambicin* promoter. M, Mutation site. **(E)** Assessment of promoter activity for STAT and Rel2 mutants. The two luciferase plasmids with M1 (STAT) and M2 (Rel2) mutants (please refer to D) were transfected into the Aag2 cells to determine the promoter activity via a luciferase assay. **(F)** Schematic representation of M3 (Rel2) and M4 (Rel1) mutants in the *Gambicin* promoter. **(G)** Assessment of promoter activity for M3 (Rel2) and M4 (Rel1) mutants. The two luciferase plasmids with M3 (Rel2) and M4 (Rel1) mutants (please refer to F) were transfected into the Aag2 cells to determine the promoter activity via a luciferase assay. **(B, E, and G)** A pAc5.1-Renilla plasmid with constitutive renilla luciferase expression was co-transfected as an internal control. The transfected cells were then challenged by *E. coli* at 0.05 OD_600_. The promoter activity in response to the bacterial infection was determined by a luciferase assay. The values of the firefly luciferase were normalized to that of the renilla luciferase. The data were analyzed using the non-parametric *Mann-Whitney* test. The data are presented as the mean ± S.E.M. Each experiment was biologically reproduced by 3 times. ^***^p < 0.0005 and ^****^p < 0.0001. n.s., no significance.

The induction of *Gambicin* was apparently reduced by interrupting either of the three pathways in the Aag2 cells (Figure 2), suggesting that *Gambicin* transcription may be combinatorially regulated by these three pathways. To investigate the regulation of *Gambicin*, we next cloned the 1000 bp promoter region which contains a TATA box and an arthropod initiator sequence (Cherbas and Cherbas, 1993) upstream of the *Gambicin* gene into a pGL3-Basic vector (Supplementary Figure S5). We designed and constructed the truncations of the *Gambicin* promoter shown in Figure 3A. A plasmid constitutively expressing renilla luciferase (pAc5.1-Renilla), which was co-transfected with the promoter-inserted recombinant plasmids, served as an internal control. The transfected cells were then challenged by *E. coli* at 0.05 OD_600_ and assayed for luciferase activation. There was no change in luciferase activation with the deletion of the −1000 to −600 bp region of the *Gambicin* promoter. However, the activity was apparently impaired by deletion of the region from −600 to −500 bp, indicating that one or more key regulatory site(s) might exist in this region (Figure 3B). In the signal cascades of the mosquito Imd, Toll, and JAK-STAT pathways, the activated of transcription factors, such as Rel1 for Toll pathway, Rel2 for Imd pathway, and STAT for JAK-STAT pathway, bind to specific regulatory sites on the promoters to initialize the transcription of downstream genes. The sequences of these regulatory sites have been characterized by previous studies (Yan et al., 1996; Ehret et al., 2001; Lin et al., 2004; Shin et al., 2005) (Figure 3C). We therefore predicted the regulatory sites within the region from −600 to −500 bp for the three pathways. Intriguingly, a STAT regulatory site (−577 to −568 bp) and a Rel2 regulatory site (−542 to −531 bp) were predicted in this region. We therefore mutated these sites in the 1000-bp *Gambicin* promoter (pGL-1k) and designated them as M1 (the STAT −577 to −568 bp mutant) and M2 (the Rel2 −542 to −531 bp mutant) (Figure 3D; Supplementary Figure S5). We then transfected luciferase plasmids with these mutants into the Aag2 cells for bacterial stimulation. Compared to the effects of the wild type promoter, both mutants significantly reduced the level of bacteria-mediated luciferase activation (Figure 3E), suggesting that both regulatory sites are independently essential for *Gambicin* induction.

Deletion of the region from −600 to −500 bp impaired the bacteria-mediated luciferase activation. Nonetheless, we did not rule out additional key regulatory sites present in the down-stream region beyond −500 bp. A potential Rel2 and a potential Rel1 regulatory sites were then predicted (−477 to −468 bp and −388 to −379 bp, respectively). We next mutated these two sites, which were designated as M3 (the Rel2 −477 to −468 bp mutant) and M4 (the Rel1 −388 to −379 bp mutant) (Figure 3F and Supplementary Figure S5). Compared to the effects of the wild type promoter, transfection by the recombinant plasmids with the M4, but not the M3, mutant repressed bacteria-mediated luciferase activation (Figure 3G), indicating that the region from −388 to −379 bp is a functional Rel1 regulatory site for *Gambicin* induction.

**Figure 4:**
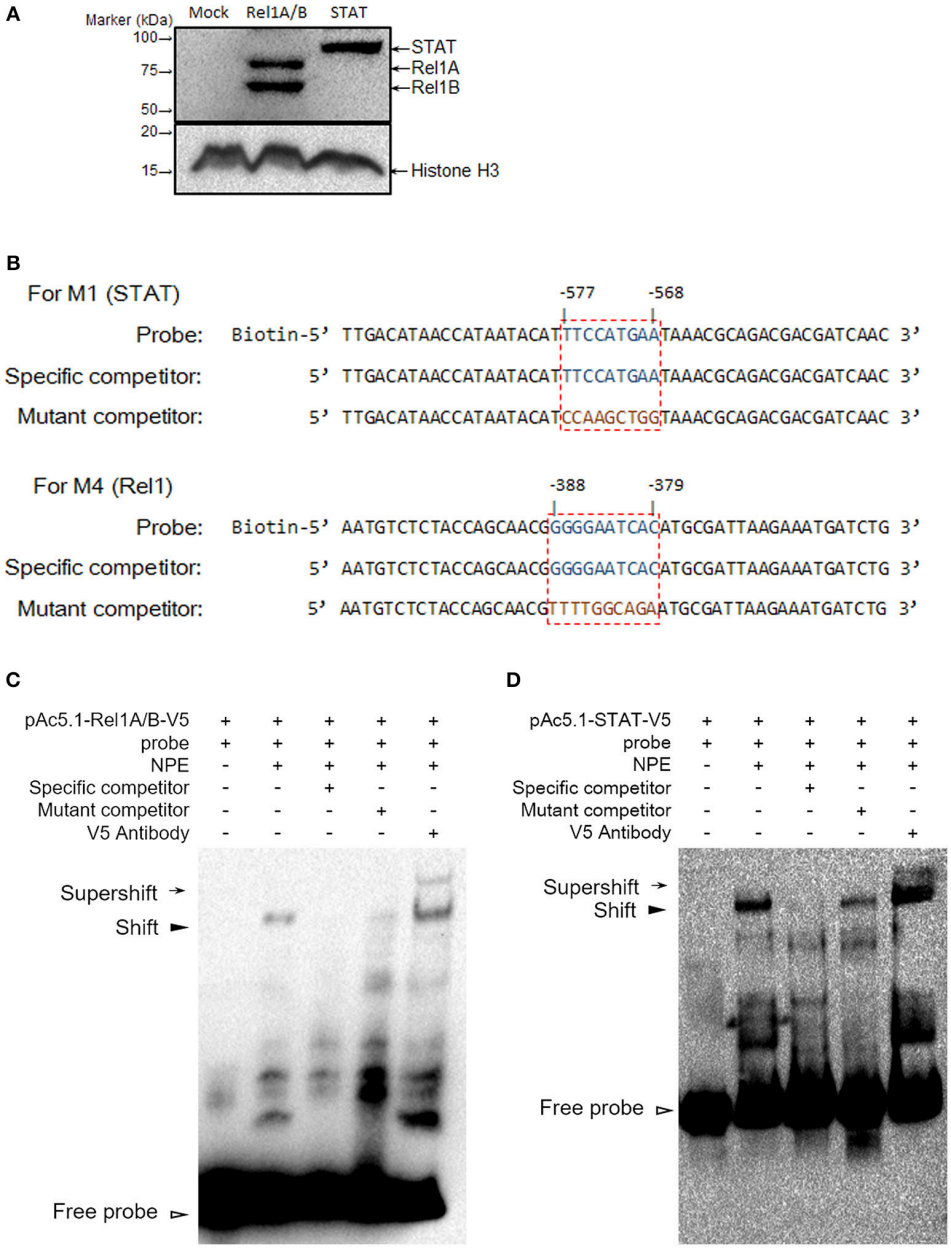
**Determine the binding affinity between Rel1 and STAT and their regulatory sites by a super-shift EMSA (A)** Ectopic expression of Rel1 and STAT in the Aag2 cells. The *A. aegypti Rel1A, Rel1B*, and *STAT* genes were cloned into pAc5.1-V5-His A vectors and designated as pAc-5.1-Rel1A-V5, pAc-5.1-Rel1B-V5, and pAc-5.1-STAT-V5, respectively. Both pAc-5.1-Rel1A-V5 and pAc-5.1-Rel1B-V5 were combined (1:1 w/w) for transfection into the Aag2 cells. The expression of Rel1A/Rel1B and STAT was confirmed by western blot analysis with an anti-V5 antibody. The detection of Histone H3 acts as an internal reference. **(B)** Design of probes for the EMSA assay. The boxed regions represent the regulatory sites for STAT and Rel1 factors. **(C,D)** Determine the binding affinity between Rel1 **(C)** and STAT **(D)** and their regulatory sites by EMSA. A V5 antibody was used to detect the specific binding between the probes and these ectopically expressed transcription factors (super-shift EMSA). The experiments were repeated 3 times with the similar results. NPE, Nuclear protein extracts.

We identified regulatory sites on the *Gambicin* promoter for transcription factors representing three signaling pathways. We next selected the *STAT* (−577 to −568 bp) and *Rel1* (−388 to −379 bp) regulatory sites to validate the binding affinity between the sequences and their transcription factors. The *A. aegypti Rel1* and *STAT* genes were cloned into pAc5.1-V5-His-A expression vectors, and subsequently, the recombinant plasmids were transfected into the Aag2 cells. The expression of *Rel1* and *STAT* was confirmed by western blot analysis with an anti-V5 antibody (Figure 4A). We silenced the *Rel2* gene by dsRNA transfection to avoid a non-specific cross-reaction between the *Rel1* regulatory site and the Rel2 factor. In the *Rel1*-transfected, *Rel2*-silenced Aag2 cells, the recognition between the *Rel1* regulatory sequence and the ectopically expressed Rel1 transcription factor was determined via a super-shift in EMSA assay (Figures 4B,C). In the *STAT*-transfected Aag2 cells, a shift of the probe at the STAT regulatory site was clearly detected using the same approach (Figures 4B,D). The V5 antibody-mediated super-shift indicated specific binding between the probes and these ectopically expressed transcription factors, demonstrating the specific recognition between the transcription factors and their regulatory binding sites.

## Discussion

Insects are equipped with multiple innate immune signaling pathways that respond to microbial invasion. Induction of *AMPs* is an important component of this systemic immune response against invading microorganisms. Generally, the Toll and Imd pathways, which are activated by microbial ligands, are reported to be intracellular immune signaling mechanisms for *AMP* induction. Our knowledge of insect *AMP* regulation has been mostly gained from investigating *Drosophila*, a general model for insect immune studies. Although phylogenetically close to *Drosophila*, mosquitoes have evolved a different *AMP* spectrum and constitution (Christophides et al., 2002; Xiao et al., 2014), which indicates that its microbe-mediated *AMP* regulation might have differences relative to that of *Drosophila*. In previous study, it was reported that *AMP* composition and antibacterial spectrum of *Anopheles gambiae* are different from those of Drosophila (Christophides et al., 2002). The results from this study provided an *A. aegypti*-specific profile for *AMP* induction in response to infections of Gram-negative bacteria (*E. coli, S. marcescens*), Gram-positive bacteria (*S. aureus, E. faecium, Leucobacter* spp.), and fungi (*C. albicans*). The *AMP* genes, especially those from the same family, were regulated by similar manners. This may be due to these genes clustered together, and shared similar binding sites for regulatory factors. For example, expression of *CecA, D, E, F, N* was induced by stimulations of *E. coli* and *S. marcescens*. And these 5 genes are clustered in a ~25 kbp area in the mosquito genome, enabling these genes sharing some analogous regulation elements. In *Drosophila*, some *AMPs* are regulated by Toll pathway, while some are regulated by Imd pathway or JAK-STAT pathway. For example, Gram negative bacteria can activate the Imd pathway, and initiate *Diptericin* expression; while Gram positive bacteria can activate the Toll pathway, and initiate *Drosomycin* expression. However, in the *A. aegypti* Aag2 cells, the Imd pathway, but not the Toll pathway, is responsible for most of the *AMP* expression induced by *E. coli*. This suggested different regulatory mechanisms of *AMP* genes between *Aedes* and *Drosophila*. And this may be an explanation of why Gram positive bacteria cannot induce the expression of *AMPs* as much as Gram negative bacteria do. Silencing the Toll pathway components, such as *Rel1A* and *MyD88*, merely impaired the transcript of one mosquito-specific *AMP, Gambicin*. In addition, the previous study indicated that the JAK-STAT pathway in *A. aegypti* responds to the up-regulation of the *Cecropin* and *Defensin* genes expressed against dengue virus infection (Souza-Neto et al., 2009). Here, we found that knockdown of the key components in the JAK-STAT pathway significantly impaired *Gambicin* responding toward bacterial infection. Deletion of a putative JAK-STAT regulatory site in the *Gambicin* promoter fully abrogated the *luciferase* activation in the *E. coli*-infected Aag2 cells, indicating that the JAK-STAT pathway is essential for the transcription of some *AMPs* in mosquitoes. Our data implicate specific innate immune signaling pathways toward the *A. aegypti AMP* expression profile operative upon bacterial infections.

The Toll and Imd pathways in *Drosophila* can combinatorially control the induction of multiple *AMP* genes, probably through the formation of heterodimers by the κB transcription factors of the Imd (Relish) and Toll (Dorsal and Dif) pathways (Hedengren-Olcott et al., 2004; Bangham et al., 2006; Lemaitre and Hoffmann, 2007; Tanji et al., 2010) or via cross recognition between κB transcription factors and their regulatory sites on the promoter (Tzou et al., 2000; Bangham et al., 2006; Lemaitre and Hoffmann, 2007), implicating the presence of confounding cross-reactions. Intriguingly, the *E. coli*-mediated induction of *Gambicin* was controlled by the Imd, Toll, and JAK-STAT signaling pathways in the Aag2 cells. Mutation of the regulatory sites for any of these three signaling pathway impaired the promoter activity, suggesting the presences of a co-regulating mechanism for microorganism-mediated *Gambicin* induction in *A. aegypti*.

*AMPs* are a family of important immune effectors in the response to microbial infections. Although their regulation has been well-established in *Drosophila*, the regulation of *AMP* expression in other insects still remains partially understood. In this study, we comprehensively examined *AMP* regulation in response to bacterial and fungal infections in *A. aegypti* embryonic-origin Aag2 cells. We further report a novel phenomenon for *AMP* regulation which can be combinatorially controlled by the NF-κB and JAK-STAT pathways. Our study adds valuable information about the regulation of *AMPs* in response to microbial infections in insects. Investigating profiles and mechanisms of *AMP* regulation in *A. aegypti* may contribute to understand the interaction between mosquitoes and their carrying pathogens.

## Author contributions

GC, RLZ and RDZ designed the experiments and wrote the manuscript; RDZ performed the majority of the experiments and analyzed data; YZ, XP and XX helped with the RNA isolation and qPCR detection. All authors reviewed, critiqued, and provided comments to the text.

## Funding

This work was supported by grants from the National Natural Science Foundation of China (81301412, 81422028, 81571975); the National Key Basic Research Program of MOST (2013CB911500). Shenzhen's Sanming Project of Health and Family planning Commission of Shenzhen Municipality. GC is a Newton Advanced Fellow awarded by the Academy of Medical Sciences and the Newton Fund, and a Janssen Investigator of Tsinghua University.

### Conflict of interest statement

The authors declare that the research was conducted in the absence of any commercial or financial relationships that could be construed as a potential conflict of interest.
