# Supplementary material for: Regulation of Antimicrobial Peptides in Aedes aegypti Aag2 Cells
Source: Front Cell Infect Microbiol. 2017 Feb 3;7:22. doi: 10.3389/fcimb.2017.00022 (PMC5291090; doi:10.3389/fcimb.2017.00022)
Supplement: Supplementary file 1 [file Presentation1.pdf]

Supplementary Material

Regulation of Antimicrobial Peptides in *Aedes aegypti* Aag2 Cells

Rudian Zhang, Yibin Zhu, Xiaojing Pang, Xiaoping Xiao, Renli Zhang* and Gong Cheng*

*** Correspondence:** Gong Cheng: gongcheng@mail.tsinghua.edu.cn; Renli Zhang: renlizhangszcdc@aliyun.com.

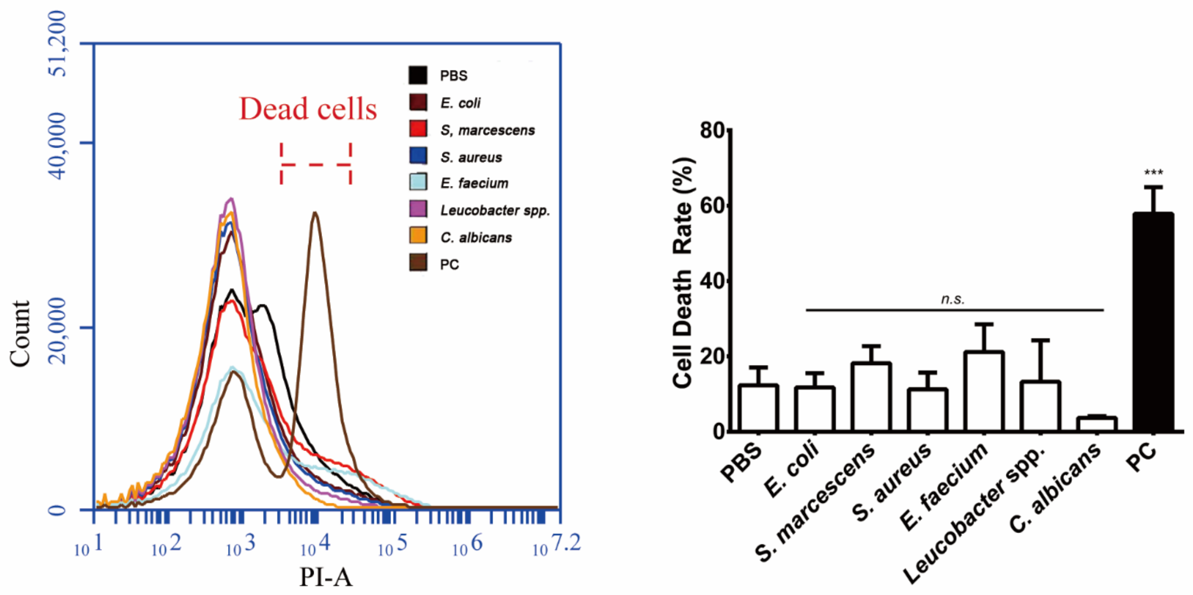

Supplementary Figure S1. Response of Aag2 cells in bacterial stimulation.

The Aag2 mosquito cells were incubated with 0.05 OD_{600} *E. coli*, *S. marcescens*, *S. aureus*, *E. faecium*, *Leucobacter* spp. and *C. albicans* cells, respectively. The Aag2 cells incubated by 20% DMSO or PBS were used as positive control (PC) or negative control, respectively. At 12 hours post microbial incubation, the stimulated Aag2 cells were collected for PI (Propidium Iodide) staining, and subsequently the dying cells were counted by flow cytometry. This experiment was repeated with 2 times with the similar result. *** P<0.0005, n.s., no significance.

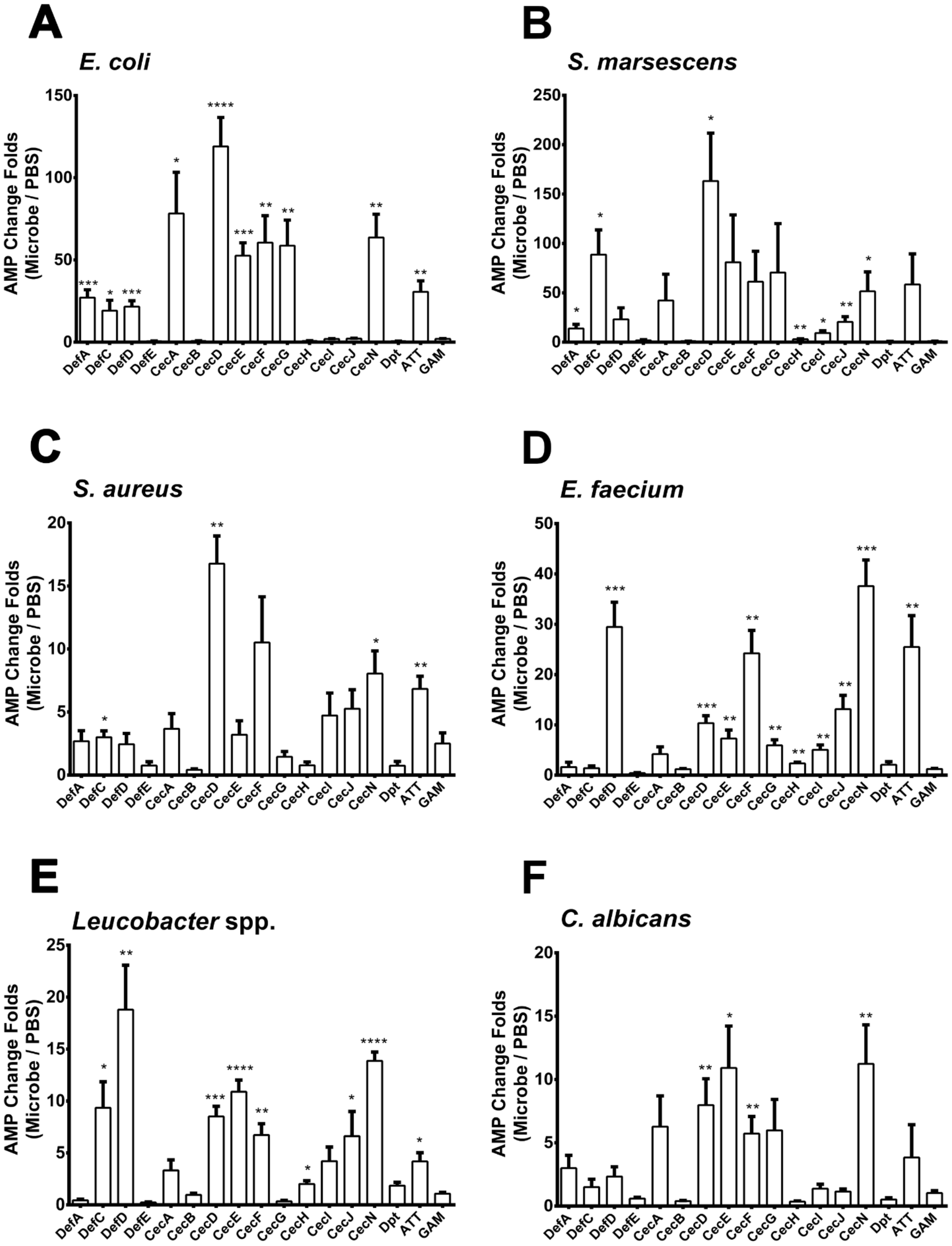
A
E. coli
B
S. marsescens
C
S. aureus
D
E. faecium
E
Leucobacter spp.
F
C. albicans
AMP Change Folds
(Microbe / PBS)
DefA
DefC
DefD
DefE
CecA
CecB
CecD
CecE
CecF
CecG
CecH
CecI
CecJ
CecN
Dpt
ATT
GAM

Supplementary Figure S2. ***AMP*** **regulation in response to microbial infection in** ***A. aegypti***

Adult female mosquitoes received a systemic injection of 300 nl of *E. coli* **(A)**, *S. marcescens* **(B)**, *S. aureus* **(C)**, *E. faecium* **(D)**, *Leucobacter spp.* **(E)** and *C. albicans* **(F)** cells at 5 OD_{600}. 6 hours later, the inoculated mosquitoes were sacrificed to isolate the total RNA for *AMP* detection. The qPCR primers for each *AMP* gene are described in Supplementary Table S2. The *AMP* stimulation is presented as the fold change relative to that in the control mosquitoes without bacterial treatment. The data are presented as the mean ± S.E.M..The differences between microbes treated groups and negative control group were analyzed by using t-test with Welch's correction. We used 6-10 mosquitoes per group in an independent experiment. The results from 2 independent experiments were combined. * $P<0.05$, ** $P<0.005$, *** $P<0.0005$, **** $P<0.0001$. Def, Defensin; Cec, Cecropin; Dpt, Diptericin; ATT, Attacin; GAM, Gambicin

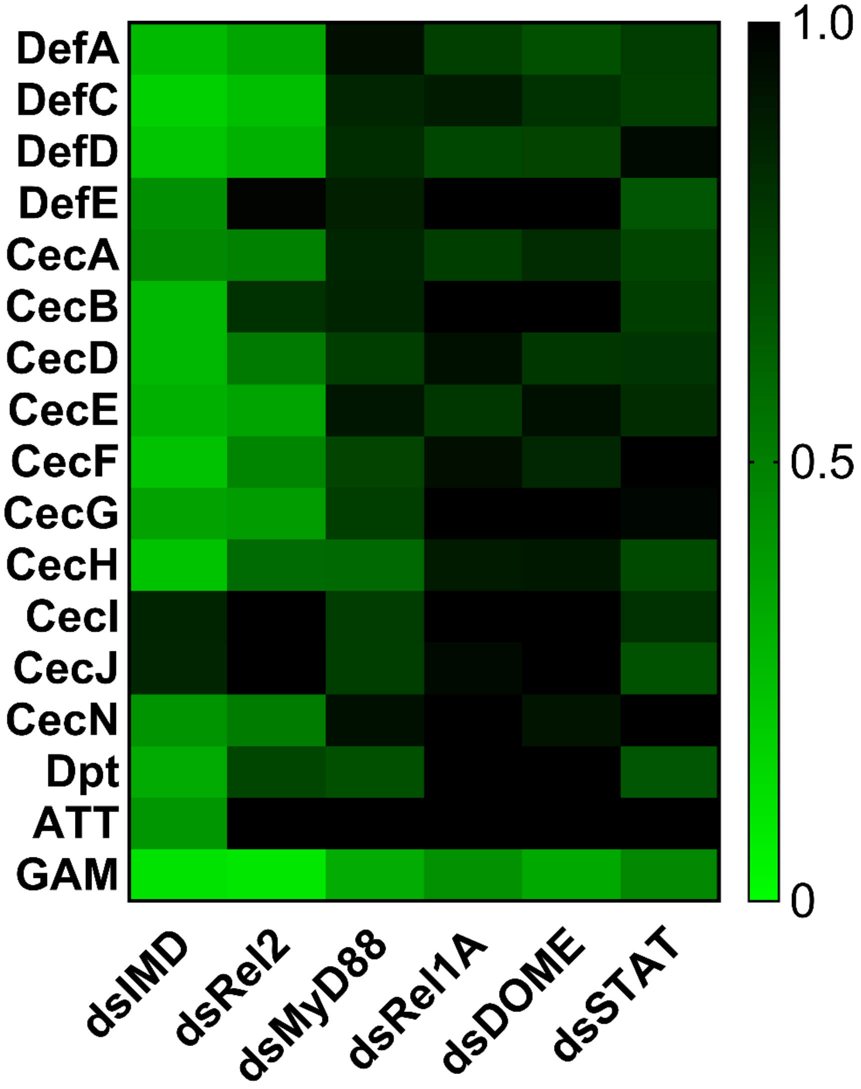

Supplementary Figure S3. Difference between negative data versus the positive data of Figure 2. Green color represents down-regulation of the *AMP* genes by genetically silencing immune components.

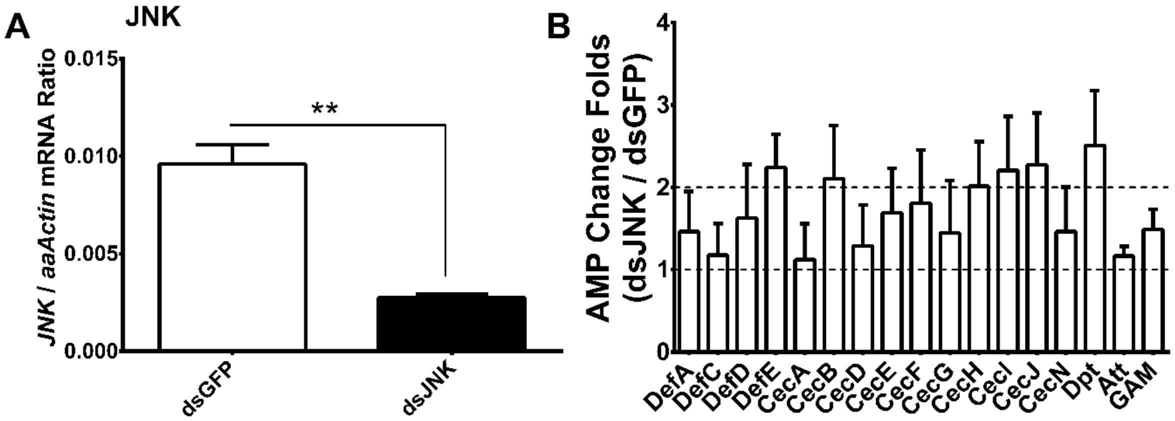

Supplementary Figure S4. Regulation of *AMPs* in *JNK* silenced Aag2 cells

(A) The knockdown efficiency of dsRNA-mediated *JNK* silencing. The gene expression was assessed through qPCR and normalized against *A. aegypti actin* (*AAEL011197*) (*aaActin*). The primers used for qPCR are described in Supplementary Table S2. The data were analyzed statistically by the t-test with Welch's correction. ** P<0.005. **(B)** The *AM*Ps regulation in microbial stimulated Aag2 cells. The data are presented as the mean ± S.E.M. The results from 2 independent experiments were combined.

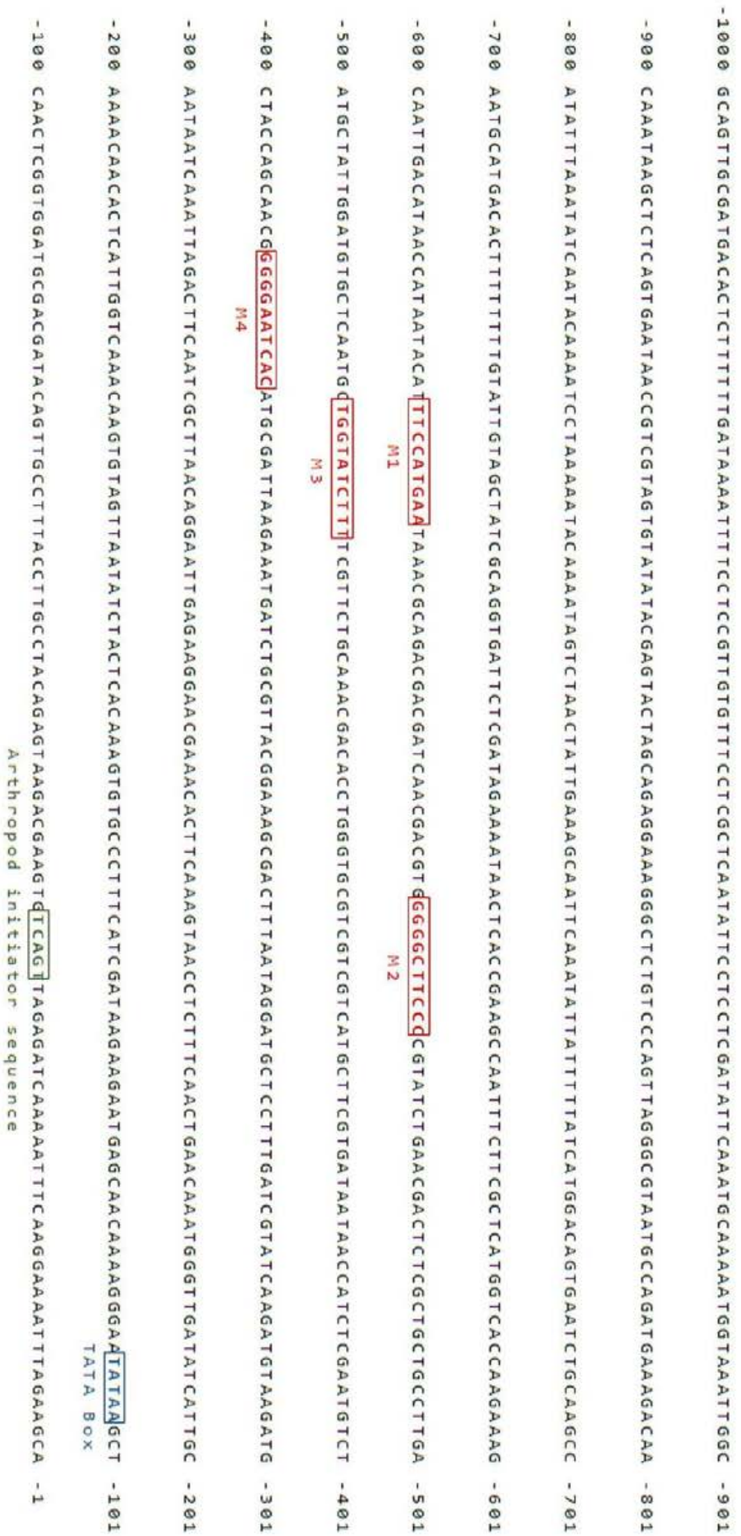

Supplementary Figure S5. Schematic representation of the Gambicin promoter region.

Supplementary Table S1. Statistic presentation of a multivariate validation of the *AMP* regulation.

Changing folds of the *AMP* genes were compared to that of *A. aegypti Actin* gene, via a two-way ANOVA analysis with Fisher's LSD test. The *p*-values in red represents significant differences.

	dsIMD	dsRel2	dsMyD88	dsRel1A	dsDOME	dsSTAT
DefA vs. *Actin*	0.0035	0.0262	0.8167	0.3374	0.1824	0.4189
DefC vs. *Actin*	0.0011	0.0099	0.5828	0.6728	0.4162	0.3925
DefD vs. *Actin*	0.0020	0.0170	0.5053	0.2760	0.2548	0.8840
DefE vs. *Actin*	0.0242	0.9391	0.6276	0.8025	0.5592	0.2423
CecA vs. *Actin*	0.0314	0.0789	0.5656	0.3507	0.4703	0.3453
CecB vs. *Actin*	0.0040	0.5149	0.5990	0.7415	0.6501	0.4007
CecD vs. *Actin*	0.0039	0.0977	0.3615	0.8049	0.3608	0.4817
CecE vs. *Actin*	0.0057	0.0270	0.7557	0.4100	0.7787	0.5468
CecF vs. *Actin*	0.0022	0.0720	0.3097	0.8131	0.5090	0.7242
CecG vs. *Actin*	0.0105	0.0354	0.3665	0.8919	0.4641	0.9148
CecH vs. *Actin*	0.0021	0.1470	0.1504	0.6933	0.6813	0.3159
CecI vs. *Actin*	0.5704	0.6955	0.3801	0.9480	0.0843	0.5181
CecJ vs. *Actin*	0.5550	0.9115	0.3598	0.8727	0.2869	0.2741
CecN vs. *Actin*	0.0188	0.0933	0.8114	0.9591	0.7328	0.5449
Dpt vs. *Actin*	0.0075	0.3421	0.2362	0.4920	0.3560	0.2424
ATT vs. *Actin*	0.0182	0.9178	0.2573	0.9061	0.5788	0.6085
GAM vs. *Actin*	0.0004	0.0018	0.0107	0.0397	0.0053	0.0404

Supplementary Table S2. Primers and probes for qPCR, dsRNA synthesis, Luciferase assay and EMSA

**Primers for cloning into pAc5.1/V5-His/A**	**Upper primer**	**Lower primer**
Rel1A(AAEL007696）	CCCTCAGGTACCATGGGACCAAACGACAACAA	CCCTCATCTAGACGGTTTGTTCAGGTTGTTGA
Rel1B(AAEL006930)	CCCTCAGGTACCATGCTCAGTCACAATCTATC	CCCTCATCTAGACTTGTTCATGTTGTTGACAGTGG
STAT(AAEL009692)	CCCTCAGGTACCATGTCGCTGTGGGCACGCGTCA	CCCTCAGCGGCCGCCCGGTGTACGACGACGCGAA
Renilla Luciferase	CCCTCAGGTACCCAGGTAAGTATCAAGGTT	TTATTGTTCATTTTTGAGAACTCGC
**The primers for RT-QPCR**	**Upper primer**	**Lower primer**
Actin(AAEL011197)	GAACACCCAGTCCTGCTGACA	TGCGTCATCTTCTCACGGTTAG
Bacteria Universal 16S rRNA	TCCTACGGGAGGCAGCAGT	GGACTACCAGGGTATCTAATCCTGTT
C. testosteroni 16s rRNA	CGAAAAGCCTGGGGCTAATAT	CCATCTCTGGTAAGTTCCTGC
C. meningosepticum 16s rRNA	ACATGGTCACCACTTCGTGAGA	GTCGCATCCGTTGTTGTCACTT
B. cereus 16s rRNA	CGCCTCGTTGGATGACG	GATATACATTCACTTGACTAATACCG
S.marcescens 16s rRNA	TTGCTCTTTAACAATCTGGAA	TCACAACCCGAAGATGTTTCC
MyD88(AAEL007768)	CGTGATTGGCGAGGGTTGTTTC	ATCCGCTCCAATGCTCGTTTCC
Rel1A(AAEL007696）	GCACCCAACAGTTCCAGAATC	GAACTGTTGTTGTTGTTGTTGCTG
Imd(AAEL010083)	TCGTCAAACTCGGTTTTCCT	TGGCGGAGTTGAAGGTAAAG
Rel2(AAEL007624)	GTTCCCGGATTAGCTACTGTG	CCGTTCCGTTTGCAATGAC
Dome(AAEL012471)	AAACGGTGGCAAAATGAACT	CATACAGCCGGCTTTCTTCT
STAT(AAEL009692)	CACACAAAAAGGACGAAGCA	TCCAGTTCCCCTAAAGCTCA
DefA(AAEL003841)	CTATCAGGCTGCCGTGGAG	CAATGAGCAGCACAAGCACTATC
DefC(AAEL003832)	CTTTGTTTGATGAACTTCCGGAG	GAACCCACTCAGCAGATCGC
DefD(AAEL003857)	GGCGTTGGTGATAGTGCTTG	CACACCTTCTTGGAGTTGCAG
DefE(AAEL003849)	GTGCGGGACACTGTCTAGCC	CAATCCTAATAATACTCATGTGCGG
CecA(AAEL000627)	CAAAGTTATTTCTCCTGATCGCG	CTGCACCTTCCAATTTCTTTCC
CecB(AAEL004223)	GCTGAAGAAGCTGGGAAAAAAG	CTTCCCAGTCCCTTGATGCC
CecD(AAEL000598)	GAAGAAGCTGGGAAAGAAATTG	CCAATCGCTTTTATTCCTACAAC
CecE(AAEL000611)	GAAAGCACTTCCCGTAGTAACTG	GTTAGTTATCACAATTCCCCCATG
CecF(AAEL000625)	GTGTTCAAAGCATCGGAAAAAG	GCTGACATTCACAATCTATCTCCG
CecG(AAEL015515)	GTTATTTCTCCTGATCGCCG	CTCGTTTTCCTGCACCTCCC
CecH(AAEL017211)	CTTCACCAAGCTGCTATTGGT	AACTTTTTTGCCAATCTTCTTCAGC
CecI(AAEL000775)	GGCTATTCGTTTTCGTCATTTTC	CTACGTTTTTGCCAGCCTTTTC
CecJ(AAEL000777)	GCTATTCGTTTTCGTCATTTTTG	CTTTTCAATCTTTTTGCCCAG
CecN(AAEL000621)	CGGCAAGAAATTGGAAAAAGTC	GAATCGATCATCCTAGGGCC
ATT(AAEL003389)	CAACACATTGCTGTTCACTTTCGT	TTGGAAGTTGTTACCTGGAGTGAG
GAM(AAEL004522)	TTTGGTGTTTGTTTATGC	GTAGCCTTTGGAGTTAAG
Dpt(AAEL004833)	GCAGCATGTGGACCAATTCA	GTTCCTTCGTCCTGTTGATGG
**The primers for double-strand RNA synthesis**	**Upper primer**	**Lower primer**
MyD88(AAEL007768)	TAATACGACTCACTATAGGGGGCGATTGGTGGTTGTTATT	TAATACGACTCACTATAGGGTTGAGCGCATTGCTAACATC
Rel1A(AAEL007696）	TAATACGACTCACTATAGGGGCACAATCTGGTCGGCAAG	TAATACGACTCACTATAGGGGGTATGCTGGAACTCGCCG
Imd(AAEL010083)	TAATACGACTCACTATAGGGAAGGCCTTCTACCAATGTAA	TAATACGACTCACTATAGGGGCACAGTGTTTTAGACTTTC
Rel2(AAEL007624)	TAATACGACTCACTATAGGGCAAGATTCGGTGTTCGCTG	TAATACGACTCACTATAGGGCCCAGTCCTGCCAGATCTC
Dome(AAEL012471)	TAATACGACTCACTATAGGGCCATCTCCACCACGAAACTT	TAATACGACTCACTATAGGGCCGGTGGTTGCCATATAATC
STAT(AAEL009692)	TAATACGACTCACTATAGGGTGGAATACCAAGGACCAAAT	TAATACGACTCACTATAGGGTGCCCAACTGCAAAGCTTGA
GFP	TAATACGACTCACTATAGGGGTGAGCAAGGGCGAGGAG	TAATACGACTCACTATAGGGCATGATATAGACGTTGTGGCTGTT
**The primers for GAM Promoter truncation**	**Upper primer**	**Lower primer**
pGL3-1k	CCCTCAGGTACCGCAGTTGCGATGACACTCTT	CCCTCACTCGAGTGCTTCTAAATTTTCCTTGA
pGL3-900	CCCTCAGGTACCCAAATAAGCTCTCAGTGAAT	CCCTCACTCGAGTGCTTCTAAATTTTCCTTGA
pGL3-800	CCCTCAGGTACCATATTTAAATATCAATACAAAATC	CCCTCACTCGAGTGCTTCTAAATTTTCCTTGA
pGL3-700	CCCTCAGGTACCAATGCATGACACTTTTTTTT	CCCTCACTCGAGTGCTTCTAAATTTTCCTTGA
pGL3-600	CCCTCAGGTACCCAATTGACATAACCATAATA	CCCTCACTCGAGTGCTTCTAAATTTTCCTTGA
pGL3-500	CCCTCAGGTACCATGCTATTGGATGTGCTCAA	CCCTCACTCGAGTGCTTCTAAATTTTCCTTGA
pGL3-400	CCCTCAGGTACCCTACCAGCAACGGGGGAATC	CCCTCACTCGAGTGCTTCTAAATTTTCCTTGA
pGL3-300	CCCTCAGGTACCAATAATCAAATTAGACTTCAATCGC	CCCTCACTCGAGTGCTTCTAAATTTTCCTTGAAATTT
pGL3-200	CCCTCAGGTACCAAAACAACACTCATTGGTCA	CCCTCACTCGAGTGCTTCTAAATTTTCCTTGA
**The primers for GAM Promoter mutation**	**Upper primer**	**Lower primer**
pGL3-M1	AACCATAATAAACGCAGACGACGATCAACG	GCGTTTATTATGGTTATGTCAATTGCTTTC
pGL3-M2	AACGCAGACGACGATCAACGACGTGGTATCTGAACGACTCTCGC	AGTCGTTCAGATACCACGTCGTTGATCGTCGTCTGCGTTTATTC
pGL3-M3	GATGTGCTCAATGCTCGTTCTGCAAACGACACCTGGGTGCGTCGT	TTGCAGAACGAGCATTGAGCACATCCAATAGCATTCAAGGCAGCA
pGL3-M4	GTCTCTACCAGCAACATGCGATTAAGAAATGATCTGCGTTACGGA	TTAATCGCATGTTGCTGGTAGAGACATTCGAGATGGTTATTATCA
**The primers for EMSA**	**Upper primer**	**Lower primer**
m1-probe	Biotin-TTGACATAACCATAATACATTTCCATGAATAAACGCAGACGACGATCAAC	Biotin-GTTGATCGTCGTCTGCGTTTATTCATGGAAATGTATTATGGTTATGTCAA
m1-specific competitor	TTGACATAACCATAATACATTTCCATGAATAAACGCAGACGACGATCAAC	GTTGATCGTCGTCTGCGTTTATTCATGGAAATGTATTATGGTTATGTCAA
m1-mutant competitor	TTGACATAACCATAATACATCCAAGCTGGTAAACGCAGACGACGATCAAC	GTTGATCGTCGTCTGCGTTTACCAGCTTGGATGTATTATGGTTATGTCAA
m4-probe	Biotin-AATGTCTCTACCAGCAACGGGGGAATCACATGCGATTAAGAAATGATCTG	Biotin-CAGATCATTTCTTAATCGCATGTGATTCCCCCGTTGCTGGTAGAGACATT
m4-specific competitor	AATGTCTCTACCAGCAACGGGGGAATCACATGCGATTAAGAAATGATCTG	CAGATCATTTCTTAATCGCATGTGATTCCCCCGTTGCTGGTAGAGACATT
m4-mutant competitor	AATGTCTCTACCAGCAACGTTTTGGCAGAATGCGATTAAGAAATGATCTG	CAGATCATTTCTTAATCGCATTCTGCCAAAACGTTGCTGGTAGAGACATT
